# Intra-articular nodular fasciitis: a rare lesion case report and an updated review of the literature

**DOI:** 10.1186/s12891-018-2375-1

**Published:** 2019-01-05

**Authors:** Wei Wang, Yiting Huang, Changxing Wang, Jianqiao Hong, Chiyuan Ma, Nong Lin, Zhaoming Ye, Shiyuan Yan, Haobo Wu

**Affiliations:** 1 Department of Orthopaedic Surgery, The Second Affiliated Hospital, Zhejiang University School of Medicine, No.88 Jiefang Road, Hangzhou, 310009 People’s Republic of China; 2 Division of Reproductive Medicine & Infertility, The Second Affiliated Hospital, School of Medicine, Zhejiang University, 88#, Jiefang Rd., Hangzhou, Zhejiang 310009 China; 3 Department of Pathology, The Second Affiliated Hospital, School of Medicine, Zhejiang University, 88#, Jiefang Rd., Hangzhou, Zhejiang 310009 China

**Keywords:** Intra-articular nodular fasciitis, Case report, Updated review of the literature

## Abstract

**Background:**

Nodular fasciitis is a benign proliferation of myofibroblasts that usually arises in subcutaneous tissues of the trunk, neck, head, and upper extremities of young adults. It is not reported to arise in the joints.

**Case presentation:**

In this report, we describe a rare case where nodular fasciitis occurred in an intra-articular location in the right knee of a 20-year-old man. The patient presented with 3-months’ duration of knee pain without history of trauma to the extremity. Physical examination revealed pain, joint effusion, and limited range of motion (ROM) of the affected knee. Magnetic resonance imaging (MRI) showed a 2.5 × 2 × 1 cm lesion in front of the posterior cruciate ligament. Arthroscopically, the soft tissue mass was removed and pathologically diagnosed as a rare, benign, intra-articular nodular fasciitis. Symptoms resolved 1 month after the operation and no recurrence was found at the 6 months follow-up.

**Conclusion:**

The present paper describes detailed characteristics of intra-articular nodular fasciitis and provides an updated comprehensive summary of 21 prior case reports.

## Background

Nodular fasciitis is a benign myofibroblastic proliferation commonly found in young adults aged between 20 and 40 years [1, 2]. It usually arises in subcutaneous tissues of the trunk, [3] neck, [4] head, [5] and the upper extremities [6]. Nodular fasciitis may also arise in the skeletal muscle, [7] dermis, [8] or in blood vessels, [9] although it is rarely reported within joints, leading to misdiagnosis. In addition to nodular fasciitis, knee pain may also arise from other diseases such as synovial chondromatosis, pigmented villonodular synovitis or giant cell tumour of the tendon sheath. We report a case of intra-articular nodular fasciitis in this study, and describe the clinical, radiological and pathological features of 21 previous case reports [10–21].

## Case presentation

A 20-year-old man presented with a history of right knee pain of 3-months duration without any trauma or undue exercise. Physical examination showed joint effusion and limited range of motion. There was no locking in the joint and no palpable mass. He had no other significant past history.

### Radiology findings

**Fig. 1:**
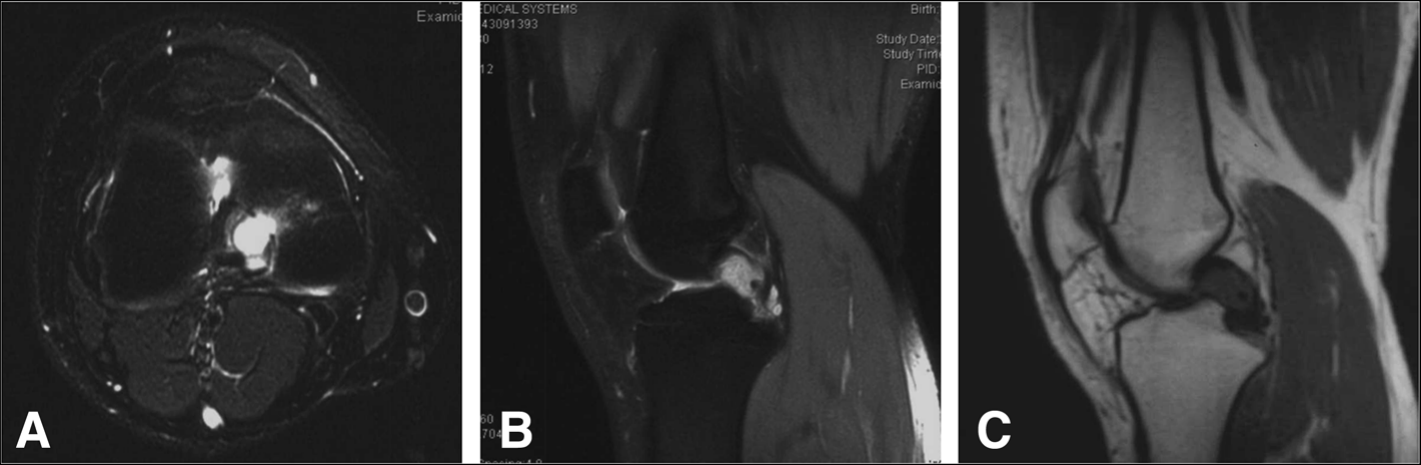
MRI of the intra-articular lesion in the right knee. **a**. T2-weighted axial MRI showed that the lesion was located between the condyles of femur with high signal intensity. **b**. T2-weighted sagittal MRI showed that the lesion was located in front of the posterior cruciate ligament with high signal intensity. **c**. T1-weighted sagittal MRI

The patient did not receive any conservative treatments. He did not receive any plain x-radiography. An MRI of the right knee showed that the intra-articular lesion was located around the posterior cruciate ligament. The lesion showed iso-intensity or lower intensity compared to surrounding muscle in T1 weighted MRIs, and high signal intensity in T2 weighted MRIs (Fig. 1). The preoperative differential diagnoses were synovial chondromatosis, pigmented villonodular synovitis or malignant soft tissue tumour. We planned to perform an arthroscopy operation to remove the lesion and to obtain a biopsy to test for malignant soft tissue tumour. If positive for malignancy, additional wide extra articular resection would be needed, and the artificial joints were prepared.

**Fig. 2:**
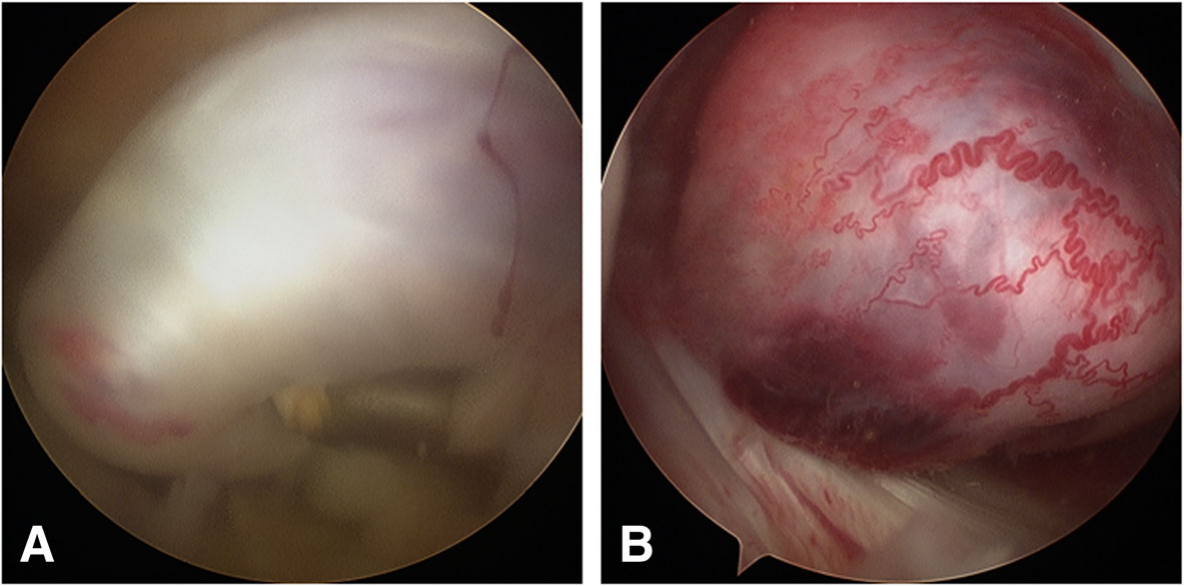
The arthroscopic view of the lesion. **a**. The lesion presented with a piece of grey-red tissue. **b**. Parts of the lesion showed blood vessels on the surface of the lesion

Therefore, arthroscopy of the right knee was performed to reveal synovial hyperplasia inflammation and the mass in front of the right posterior cruciate ligament.(Fig. 2) The lesion was excised, and partial synovectomy was performed.

### Pathology findings

**Fig. 3:**
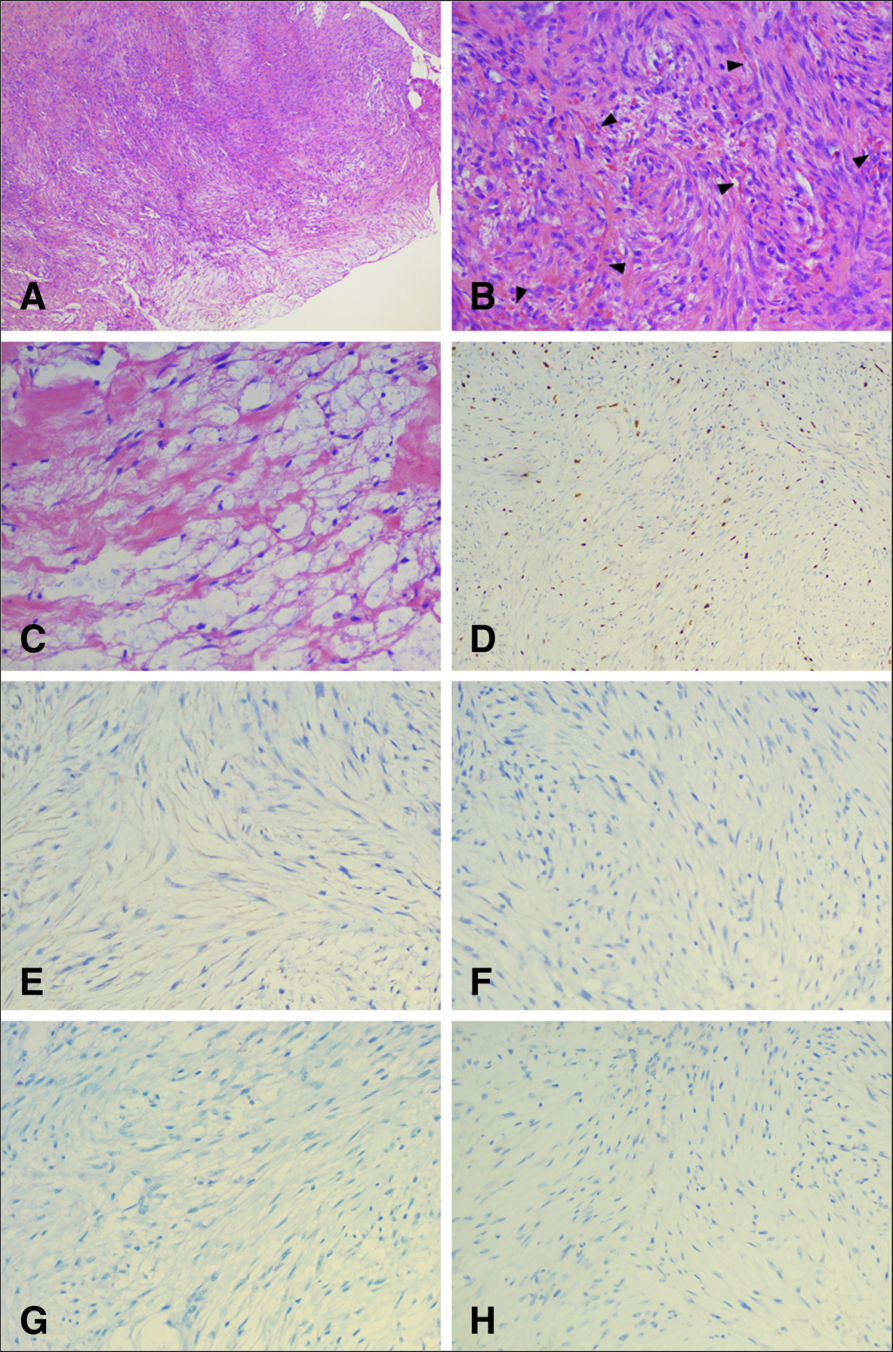
The pathological findings of the intra-articular lesion. **a**. Low-magnification (haematoxylin and eosin, original magnification × 40) image showed the unencapsulated and well-circumscribed tumour. **b**. The tumour consists of a bland fibroblastic proliferation arranged in irregular fasciitis with tissue-culture-like appearance. Extravasated erythrocytes, shown by arrows, are presented throughout the lesion (haematoxylin and eosin, original magnification × 200). **c**. The stroma varies from focally myxoid with microcyst formation to collagenous (haematoxylin and eosin, original magnification × 200). **d**. Ki-67 was 10% (original magnification × 100). **e**. Patchy positive for smooth muscle actin (original magnification × 200). **f**. Negative for desmin (original magnification × 200) **g**. Negative for S100 (original magnification × 200). **h**. Negative for CK(AE1/AE3) (original magnification × 200) in lesion cells

Macroscopically, the right knee mass presented with a piece of grey-red tissue measuring 2.5 cm by 2 cm by 1 cm in size. The antibodies, clones, dilutions, pretreatment conditions, and sources are listed in Table 1. On microscopic examination in Fig. 3, the tumour consisted of a bland fibroblastic proliferation arranged in irregular fasciitis with tissue-culture-like appearance. The stroma varied from focally myxoid with microcyst formation to collagenous. Extravasated erythrocytes and small lymphocytes were present throughout the lesion. No areas of necrosis or atypical mitosis were seen. Immunohistochemistry in Fig. 3 demonstrated that the cells were positive in patches for SMA, and negative for S100, desmin, CK(AE1/AE3), nuclear stain of beta catenin and CD34 in lesion cells. Ki-67 stained 10% of cells. According to clinical features, imaging and histology, the final diagnosis was intra-articular nodular fasciitis, which is usually a self-limiting and regressing fibrous process. Recurrence after incomplete excision has been occasionally observed.

**Table 1:** Details of Antibodies Used in this Study

Antigen	Clone	Dilution	AntigenRetrieval	Source
SMA	1A4	1:200	None	Sigma,St.Louis,MO
S100 protein	15E2E2 + 4C4.9	1:600	None	Dako
desmin	EP15	1:100	None	Dako
CK(AE1/AE3)	AE1/AE3	1:300	None	Sigma,St.Louis,MO
beta-catenin	UMAB15	1:800	None	Dako
CD34	QBEnd/10	1:800	None	Genetex
Ki-67	MIB-1	1:300	None	Abcam
P53	DO-7	1:400	None	Santa Cruz

*SMA* smooth muscle actin, *CK* Cytokeratin

### Follow-up

The symptoms of painful joint effusion and limited range of motion were improved 1 month after the operation. No recurrence was observed at the 6-months’ follow-up.

## Discussion

### Clinical findings and radiological findings

The clinicopathological findings are summarized in Table 2. There were 21 cases of intra-articular nodular fasciitis from 12 studies. Nine patients were female and 12 were male. The age at diagnosis ranged from 4 to 54 years old, with a median of 26 years. Twelve patients presented in the second to forth decades of life. The duration of symptoms before surgical excision ranged from 1 month to 1 year (median, 4 months). Fourteen lesions arose in the knee, 3 in the shoulder, 2 in the hand, 1 in the hip, and 1 in the ankle. Only 5 patients, including those with 2 lesions in the shoulder, 2 in the knee and 1 in the ankle, reported prior trauma. Most patients presented with a painful mass and limited range of motion. Eight patients came to the outpatient clinic for the palpable mass. The T1-weighted MRI revealed iso-signal intensity or lower signal intensity compared to the surrounding normal muscle, while the T2-weighted MRI showed high signal intensity and pronounced high signal intensity.

**Table 2:**
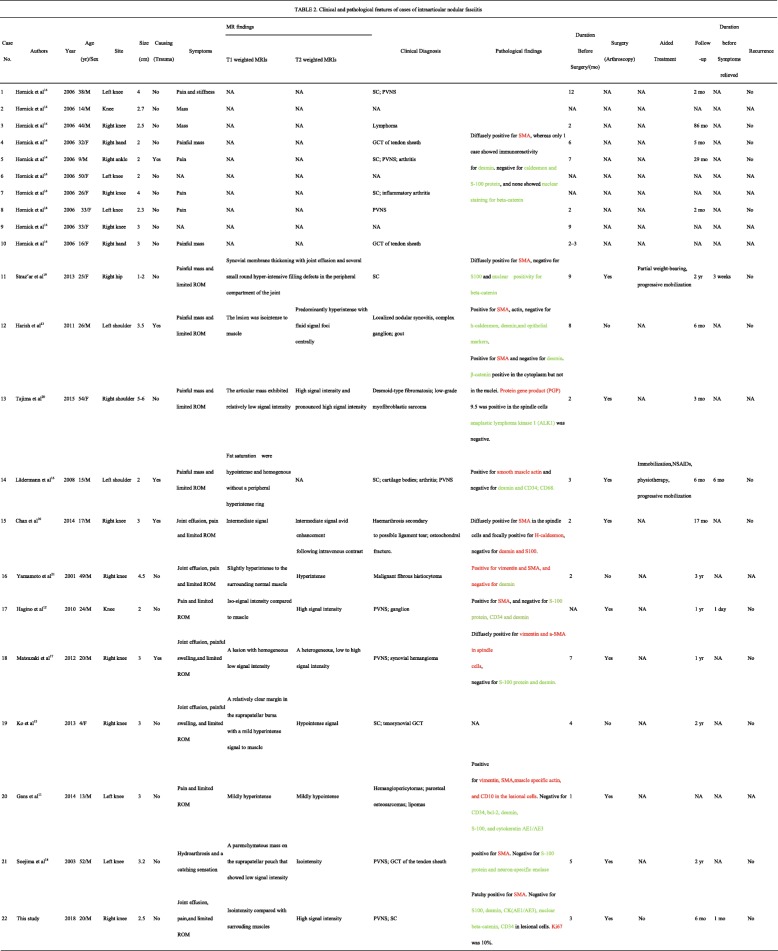
Clinical and pathological features of cases of intraarticular nodular fasciitis

*NED* no evidence of disease, *NA* not available, *PVNS* pigmented villonodular synovitis, *GCT* giant cell tumor, *SC* Synovial chondromatosis, *ROM* range of motion, *yr* years, *mo* months, *SMA* smooth muscle actin, *CK* Cytokeratin, *NSAIDs* non-steroidal antiinflammatory drugs.The red color indicates the positive marker, while the green color indicates the negative marker in the pathological findings

Clinical differential diagnoses included synovial chondromatosis (7 cases), pigmented villonodular synovitis (7 cases), giant cell tumour (4 cases), inflammatory arthritis (3 cases), complex ganglion (2 cases), lymphoma (1 case), gout (1 case), desmoid-type fibromatosis (1 case), low-grade myofibroblastic sarcoma (1 case), cartilage bodies (1 case), haemarthrosis (1 case), osteochondral fracture (1 case), malignant fibrous histiocytoma (1 case), haemangioma (1 case), lipomas (1 case), haemangiopericytomas (1 case), parosteal osteosarcomas (1 case), localized nodular synovitis (1 case), and fibromyxoid sarcomas (1 case). Three of 21 cases did not undergo arthroscopic surgery. The duration of follow-up ranged from 2 months to 86 months. No recurrence was observed.

### Macroscopic features

Lesion sizes ranged from 1 to 6 cm (median, 3 cm) in the largest dimension. All tumours were a solid mass in gross appearance, usually grey or yellow.

### Microscopic and immunohistochemical features

Histologic features showed typical nodular fasciitis, which was composed of cytologically bland and uniform plump spindle cells. The spindle cells were arranged within a variably loose myxoid to collagenous stroma in the form of short intersecting bundles. The loose myxoid to collagenous stroma contained scattered lymphocytes and red blood cells.

By immunohistochemistry, all cases showed that the spindle cells were diffusely positive for alpha-smooth muscle actin (SMA). All cases except one were negative for desmin. All cases except one were negative for caldesmon. None showed positive for nuclear beta-catenin and S-100 protein. One case in the shoulder showed positive for protein gene product (PGP) and negative for anaplastic lymphoma kinase 1 (ALK1) in the spindle cells. Another case in the shoulder showed negative for CD34 and CD68. Three cases in the knee showed positive for vimentin in the spindle cells. One case in the knee showed positive for muscle-specific actin, CD10 and negative for bcl-2 and cytokeratin AE1/AE3.

### Follow-up

Only three cases, including one shoulder and two knees, did not undergo arthroscopic surgery to excise the lesion. Most patients’ symptoms were relieved a few days after surgery. For the patient with the lesion in the hip, partial weight-bearing on crutches and progressive post-operative mobilization were recommended, and the patient’s symptoms were relieved 3 weeks after surgery. Another case in the knee was recommended immobilization and non-steroidal anti- inflammatory drugs (NSAIDs) for 10 days, followed with physiotherapy with progressive mobilization; the patient’s symptoms were relieved 6 months after surgery. No recurrences were reported in any follow-up.

Intra-articular nodular fasciitis is rarely reported. Until now, only 21 cases of intra-articular nodular fasciitis have been documented in the literature (Table 2). To the best of our knowledge, only 1 previous clinicopathologic analysis of a series of intra-articular nodular fasciitis cases was reported, which only included 7 cases in knees, 2 cases in hands and 1 case in the ankle. In this present report, we described the case of intra-articular nodular fasciitis in the knee of a 20-year-old Chinese man and updated the case series of intra-articular nodular fasciitis, including 14 cases in knees, 3 cases in shoulders, 2 cases in hands, 1 case in the ankle and 1 case in the hip.

The clinicopathological features are summarized in Table 2. Most cases presented during the first to fifth decades of life, some with and some without trauma. The clinical history of patients with intra-articular nodular fasciitis is as short as 1 month. The lesions ranged from 1 to 6 cm. Radiologically, the lesions showed iso-signal intensity or lower signal intensity compared to muscle in T1-weighted and hyper-intensity T2-weighted MRIs. The follow up showed no recurrences, indicating it was a benign course.

It was usually misdiagnosed because of its rare incidence rate. Most cases were clinically misdiagnosed to be synovial chondromatosis (7 cases), pigmented villonodular synovitis (7 cases), giant cell tumour of tendon sheath (4 cases) or desmoid-type fibromatosis (1 case). These possibilities are excluded by histologic examinations. Histologically, the lesions showed typical nodular fasciitis, which is composed of cytological bland and uniform, plump spindle cells. The spindle cells were arranged within a variably loose myxoid to collagenous stroma in the form of short, intersecting bundles.

Desmoid-type fibromatosis is an abnormal growth that arises in the connective tissue, including abdominal wall, shoulders, upper arms, and upper legs [22]. It is aggressive and can recur easily. Desmoid-type fibromatosis consists of sweeping fascicles of uniform, fibroblastic cells within a collagenous stroma. Blood vessels are often small and compressed in the lesion. Hyalinized or keloidal-type collagen fibres can usually be observed [22]. Some studies indicated that nuclear beta-catenin by immunohistochemistry may be useful in differential diagnosis [22–24]. Additionally, the Ki67 proliferative index may also be useful for distinguishing nodular fasciitis from desmoids tumour [25].

Pigmented villonodular synovitis (PVNS, diffuse-type giant cell tumour) and giant cell tumour of tendon sheath (localized giant cell tumour of tendon sheath) were included in the giant cell tumours (GCT) of the synovium and tendon sheath [26]. Histological examination reveals mononuclear stromal cell infiltrate involving synovial membrane, haemosiderin-laden macrophages, foam cells and multinucleated giant cells [27].

Synovial chondromatosis is determined by histological evaluation. The number of nodules in synovial chondromatosis can be counted in the thousands. Microscopically, the nodules are composed of hyaline cartilage with synovial tissue lining on the outside [28]. The chondrocytes can show mild atypia, myxoid changes, calcification, or ossification [29].

Tendon sheath fibroma should also be distinguished from nodular fasciitis, because tendon sheath fibromas share most of the immunohistochemical markers with nodular fasciitis, such as positive vimentin, smooth muscle actin and negative desmin [30]. However, tendon sheath fibroma is characterized by spindle-shaped and stellate-shaped fibroblasts, a fibrocollagenous, partly myxoid stroma, and slit-like vessels in histologic features [30]. The presence of a less orderly, tissue culture-like growth pattern, extravasated red blood cells and more prominent myxoid stroma favours the diagnosis of nodular fasciitis [14].

Intra-articular fasciitis presented with some distinctive features from extra-articular fasciitis. On one hand, prominent stromal hyalinization is quite common in intra-articular lesions caused by repeated, frictional trauma. On the other hand, haemosiderin deposition is also frequently seen in tissues adjacent to intra-articular nodular fasciitis secondary to trauma.

Intra-articular nodular fasciitis, as in the musculoskeletal disorders, is usually identified with MRI. On the other hand, it was reported that ultrasound is also helpful in identifying the intra-articular nodular fasciitis [31]. For musculoskeletal disorders, ultrasound is applied widely in evaluating dynapenia [32] and guiding subacromial corticosteroid injection [33].

## Conclusions

In summary, nodular fasciitis can occur in the joints, most frequently in the knees and shoulders, without gender preference, in patients between 10 to 50 years old. It generally has a longer preoperative history than other cases of nodular fasciitis. The lesions show iso-signal or lower intensity compared to muscle in T1-weighted and hyper-intensity T2-weighted MRIs. The histological features are typical of nodular fasciitis, consist of a bland fibroblastic proliferation arranged in irregular fasciitis with tissue-culture-like appearance. It appears not to recur, though the number of reported cases is limited. Awareness of the occurrence of nodular fasciitis within joints including knees, shoulders, hands, ankle and hip will lead to the correct diagnosis. Additional reports about intra-articular nodular fasciitis cases in the elbow and other joints are needed in the future.

## Abbreviations

CK: Cytokeratin
GCT: giant cell tumour
mo: months
NA: not available
NED: no evidence of disease
NSAIDs: non-steroidal anti-inflammatory drugs
PVNS: pigmented villonodular synovitis
ROM: range of motion
SC: Synovial chondromatosis
SMA: smooth muscle actin
yr.: years
